# CD81 and Hepatitis C Virus (HCV) Infection

**DOI:** 10.3390/v6020535

**Published:** 2014-02-06

**Authors:** Lucie Fénéant, Shoshana Levy, Laurence Cocquerel

**Affiliations:** 1 Center for Infection and Immunity of Lille, CNRS-UMR8204, Inserm-U1019, Institut Pasteur de Lille, Université Lille Nord de France, Institut de Biologie de Lille, 1 rue du Pr Calmette, CS50447, 59021 Lille Cedex, France; E-Mail: lucie.feneant@ibl.fr; 2 Department of Medicine, Division of Oncology, CCSR, Stanford University Medical Center, Stanford, CA 94305, USA; E-Mail: slevy@stanford.edu

**Keywords:** Hepatitis C Virus, CD81, tetraspanins, entry factors, viral lifecycle, immune response

## Abstract

Hepatitis C Virus (HCV) infection is a global public health problem affecting over 160 million individuals worldwide. Its symptoms include chronic hepatitis, liver cirrhosis and hepatocellular carcinoma. HCV is an enveloped RNA virus mainly targeting liver cells and for which the initiation of infection occurs through a complex multistep process involving a series of specific cellular entry factors. This process is likely mediated through the formation of a tightly orchestrated complex of HCV entry factors at the plasma membrane. Among HCV entry factors, the tetraspanin CD81 is one of the best characterized and it is undoubtedly a key player in the HCV lifecycle. In this review, we detail the current knowledge on the involvement of CD81 in the HCV lifecycle, as well as in the immune response to HCV infection.

## 1. Introduction

In 1990, the target of an anti-proliferative antibody was identified as a 26 kDa cell surface protein expressed on most human cells [1]. This protein was first called TAPA-1 for target of anti-proliferative antibody 1 and, following the Fifth International Workshop on Human Leukocyte Differentiation Antigens, it has been renamed CD81. It was subsequently demonstrated that CD81 is involved in an astonishing number of cellular processes such as adhesion, morphology, activation, proliferation and differentiation of immune cells (reviewed in [2,3,4,5]). Moreover, CD81 is also involved in infection by many pathogens including parasites, bacteria, fungi and viruses (reviewed in [6]). Among them, Hepatitis C Virus (HCV) is strictly dependent on CD81 to initiate its entry into its target cells, the hepatocytes.

HCV infection is a global public health problem with 160 million individuals infected [7]. An estimated additional two million people are newly infected per year, most of them through contaminated needle injections [8]. Only few patients clear the virus spontaneously and up to 80 % of HCV infected people become chronically infected. Chronic infection leads to hepatic steatosis, cirrhosis and hepatocellular carcinoma [9] and represents the major reason for liver transplantation. Until recently, the standard-of-care (s.o.c.) therapy was based on a combination of pegylated interferon-α and ribavirin, [10]. However, it was not efficient on all HCV genotypes and limited by drug resistance, toxicity and high costs. The most recent addition of protease inhibitors (Boceprevir and Telaprevir) to s.o.c. therapy has significantly improved the efficacy of treatment, especially for HCV genotype 1-infected patients, which are the most resistant to the s.o.c. therapy [11,12]. Moreover, new direct acting agents (DAAs) have been approved and are expected in the next months. However, the absence of a preventive vaccine, the sustained number of non-responsive patients to current treatments and the high cost of upcoming DAAs make the search for new treatments essential. 

**Figure 1:**
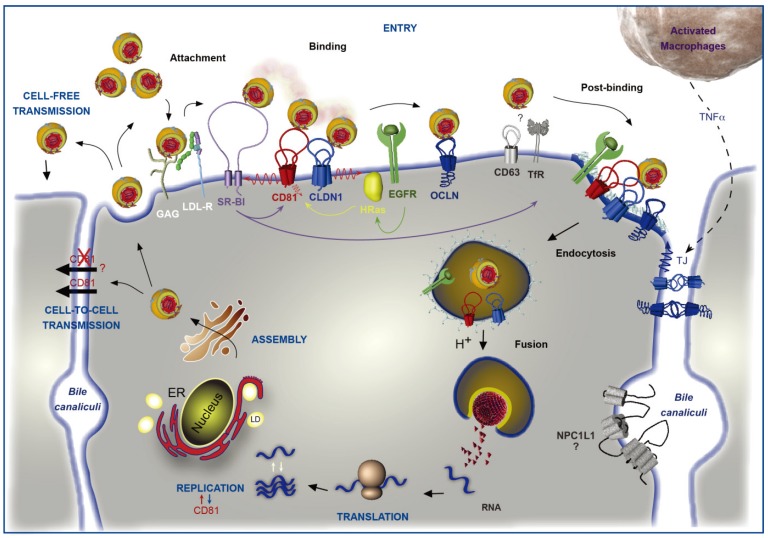
Involvement of CD81 in Hepatitis C Virus (HCV) lifecycle. HCV initiates its infection into hepatocytes by an attachment step at the cell surface in which virions interact with non-specific factors such as glycosaminoglycans (GAG). Due to the association of viral particles with lipoproteins, the Low Density Lipoprotein-Receptor (LDL-R) likely plays a role in this initial step of entry. Then, viral particles bind to specific entry factors including CD81, which occupies a central position in the entry factor complex and which interplays with its partners. HCV first interacts with the scavenger receptor class B type I (SR-BI), which in turn probably facilitates the association of viral envelope proteins with CD81. CD81 and the tight junction protein claudin-1 (CLDN1) naturally form a complex that is essential to HCV entry and which is likely regulated by the Epidermal Growth Factor-Receptor (EGFR) and the GTPase HRas. After interaction with the CD81/CLDN1 complex, HCV interacts with occludin (OCLN), another tight junction protein. Other molecules, such as the transferrin receptor (TfR), the tetraspanin CD63, and the Niemann-Pick C1-like1 (NPC1L1) cholesterol transporter, which is mainly localized in bile canaliculi (BC), have been shown to also be involved in HCV entry but for which mechanisms need to be elucidated. The membrane diffusion of CD81 (depicted by the red ↯) is another important element regulating HCV entry. The virus is next internalized by clathrin-mediated endocytosis, possibly in association with CD81/CLDN1 complex and EGFR. Internalization is likely favored by the lipidic transfer properties of SR-BI. After fusion at low pH with the membrane of an early endosome, the viral genome is released into the cytosol. Next, translation and polyprotein processing take place and the viral RNA is replicated. It has been shown that CD81 could be involved in the process of replication and conversely RNA replication could regulate CD81 expression levels. In the late stages of the cycle, new virions are assembled in an ER-related compartment in close connection with the Very Low Density Lipoproteins (VLDL) biogenesis pathway. This process seems to occur in the proximity of lipid droplets (LD). Virions that are released can infect new cells by cell-free transmission. Particles can also be transferred directly to the neighboring cells by cell-to-cell transmission for which CD81-independent and CD81-dependent routes have been described but are still controversial. Very recently, it has been shown that activated macrophages produce TNFα that increases the diffusion coefficient of CD81 and relocalizes OCLN at the basolateral membrane, thereby facilitating HCV entry.

The selective association of a virus with a target cell is usually determined by an interaction between the viral glycoproteins and specific cell–surface receptor(s) and is an essential step in the initiation of infection. Such interaction(s) often define the host range and cellular or tissue tropism of a virus and have a role in determining virus pathogenicity. HCV infection begins with the attachment of the viral particle to the cell surface of the hepatocytes through attachment factors such as glycosaminoglycans (GAG) and Low Density Lipoproteins Receptor (LDL-R) [13,14,15] (Figure 1). This preliminary attachment allows the contact between the viral particle and a series of specific cell entry factors, including the tetraspanin CD81, which is the first to have been identified [16], and is the best characterized entry factor for HCV. The Scavenger Receptor class B type 1 (SR-BI) [17], the tight junction proteins claudin-1 (CLDN1) [18] and occludin (OCLN) [19,20], the tyrosine kinase receptors EGFR and Ephrin A2 [21] and the cholesterol uptake receptor Niemann-Pick C1-like 1 [22] receptor were also described as HCV entry factors. More recently, CD63, another tetraspanin family member [23] and the transferrin receptor 1 [24] have also been described as entry factors. The interaction of HCV particles with these different entry factors leads to the internalization of the particle through a clathrin-mediated endocytosis [25,26] and to its fusion at low pH with the membrane of an early endosome [27,28]. The viral RNA is then released into the cytosol where it is translated into a polyprotein, which is maturated into structural and non-structural (NS) proteins, while NS3 to NS5B proteins constitute the replicase complex leading to the synthesis of new genomic RNAs [9]. Subsequently, HCV particles are assembled in close connection with the Very Low Density Lipoproteins (VLDL) pathway [29] and are released from cell through the secretory pathway. Next, new cells can be infected either from newly released free HCV particles, or directly from cell-to-cell transmission (Figure 1). 

Although it has been largely demonstrated that CD81 plays a key role in HCV entry process, it has been demonstrated that this tetraspanin is also likely involved in HCV replication and immune response to HCV infection. In this review, we detailed the current knowledge on the involvement of CD81 in HCV infection.

## 2. HCV Particle and Model Systems to Study the HCV Lifecycle

**Figure 2:**
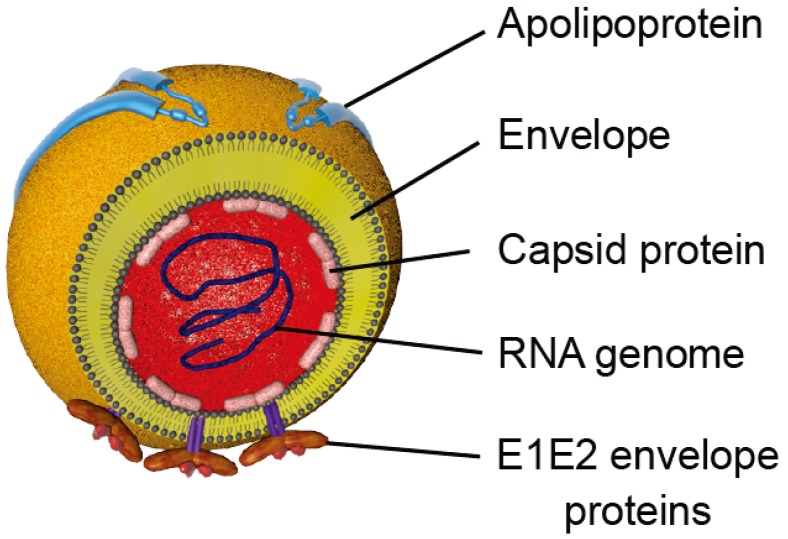
Schematic representation of HCV particles. Viral particles are composed of a nucleocapsid containing the viral RNA surrounded by a host cell-derived lipid envelope in which the E1 and E2 envelope glycoproteins are embedded. Apolipoproteins that are associated with particles are represented.

HCV is a small enveloped virus belonging to the *Hepacivirus* genus in the *Flaviviridae* family (reviewed in [30]). Its genome is a positive single strand RNA encoding a polyprotein of approximately 3000 amino acids. This polyprotein is cleaved by cellular and viral proteases into structural (E1, E2 and Core) and non-structural (p7, NS2, NS3, NS4A, NS4B, NS5A, NS5B) proteins (reviewed in [31]). The viral particle is composed of a nucleocapsid protecting the viral RNA, surrounded by a lipidic cell-derived envelope in which the glycoproteins E1 and E2 are embedded (Figure 2). It has to be noted that HCV virion is tightly associated with lipoproteins to form a hybrid particle that has been called lipoviroparticle (LVP) and lipoprotein components are involved in HCV entry (reviewed in [32]).

For a long time, there was no cell culture system available to study HCV entry; whereas replication of subgenomic HCV RNAs was demonstrated early on [33,34]. Recombinant soluble truncated forms of E2 (sE2) were first used to identify HCV entry factors [35,36]. However, these soluble proteins did not fully mimic E2 on the viral particle where it is assembled with E1, in the E1E2 complex [37,38,39,40,41]. The development of lentiviral particles pseudotyped with HCV glycoproteins (HCVpp), allowed for the first time the study of all steps of HCV entry [28,42]. However, the HCVpp system did not completely simulate HCV entry because 293T cells, which are used to produce HCVpp, do not allow the association of particles with lipoproteins. Indeed, HCV particle assembly is closely associated with the VLDL pathway in hepatocytes [29,43] resulting in the incorporation of some apolipoproteins (Apo) into particles, including ApoE, ApoC1, ApoB and ApoA-I [32,44,45]. The most important milestone in HCV research was the development of a cell culture system that enables efficient *in vitro* amplification of HCV [30,46,47]. These particles named HCVcc, for cell culture derived HCV, are produced by transfecting the human hepatoma Huh-7 cell line with a HCV genome isolated from a patient with a fulminant hepatitis C (JFH-1). HCVcc particles are infectious in hepatocyte-derived cell lines, primary cells as well as in animal models and allow the dissection of the entire HCV lifecycle.

## 3. CD81 Plays a Major Role in HCV Entry

Because HCV entry is required for initiation, dissemination and maintenance of viral infection, it is a promising target for antiviral therapy. Although many advances have been made in recent years, little is known about the precise role of the different cellular entry factors involved in HCV entry. It is acknowledged that HCV entry is an intricate multistep process, which is likely mediated through the formation of a tightly orchestrated HCV entry factor complex at the plasma membrane of the hepatocytes. However, the interaction kinetics still need to be exactly defined. Anyway, since its identification in 1998 as the first putative receptor for HCV [16], CD81 has been demonstrated to be a key player in HCV entry and is by far the best characterized of the cellular entry factors. 

**Figure 3:**
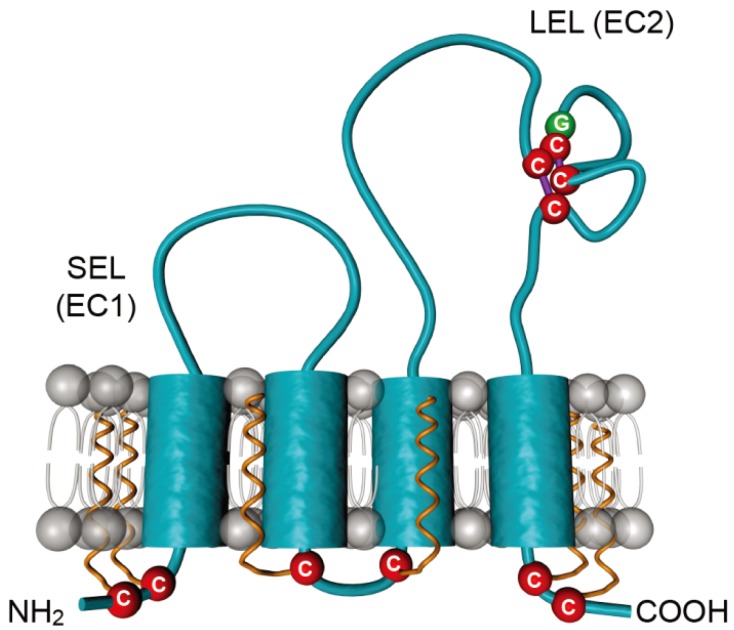
Schematic representation of the tetraspanin CD81. CD81 is composed of four transmembrane domains and two extracellular loops designated the small extracellular loop (SEL or EC1) and the large extracellular loop (LEL or EC2). Conserved cysteines are highlighted in red. The conserved CCG motif, which forms disulfide bridges (purple lines) with additional cysteines, is shown. Palmitoylations on six juxtamembranous cysteines are shown in orange.

CD81 is a 236 amino acid protein, which protrudes just 3.5 nanometers of the membrane bilayer [48]. It is a member of the tetraspanin family, which is characterized by four transmembrane segments linked by one short extracellular (SEL), one short intracellular and one large extracellular (LEL) stretches (Figure 3). CD81 is also characterized by four conserved cysteine residues, including an ubiquitous CCG motif and two additional cysteines in the LEL that form critical disulfide bonds in the LEL. In contrast to other tetraspanins, CD81 is not N-glycosylated but it undergoes palmitoylation on six juxtamembranous cysteine residues [49,50]. CD81 is ubiquitously expressed, except in red blood cells and platelets. In the liver, it is expressed both on sinusoidal endothelium and on hepatocytes, where it is mainly localized in the basolateral membrane [51].

Three lines of CD81 knockout mice (*Cd*81KO) have been independently-derived and have impairments in their immune system, which is likely due to cell-to-cell miscommunications [52,53,54]. A more distinct example of the effect of lack of CD81 on cell–cell communication is demonstrated by the inability of *Cd*81KO eggs to be fertilized by sperm, leading to female infertility [55]. A recent study demonstrated impairment in muscle regeneration in *Cd*81KO mice [56]. Finally, it is noteworthy that CD81 is also required for the lifecycle of another major human pathogen, *Plasmodium*, the malaria-causing parasite. *Cd*81KO mice are resistant to infection by *P. yoelii* sporozoites, the liver stage of the parasite lifecycle. Moreover, anti-human CD81 antibodies blocked infection of human hepatocytes by *P. falciparum*, the human pathogen [57]. However, CD81 is not a receptor for this pathogen, as it does not bind sporozoites directly [58]. Taken together, lack of CD81 impedes normal cell–cell interactions, which are possibly usurped by HCV.

### 3.1. CD81, a Key Player in HCV Entry

Since its identification as a molecule that interacts with sE2 [16], the involvement of CD81 in HCV entry has been confirmed in numerous studies. Indeed, antibodies directed against the LEL of CD81 are able to inhibit entry of HCVpp, HCVcc and serum-derived HCV [15,28,46,47,59,60,61,62,63,64] and HCV infection *in vivo* [65]. Although the affinity of E2 glycoprotein for CD81 [66] may differ depending on viral genotype [67,68,69,70], anti-CD81 antibodies are able to block infection of HCV from different genotypes [63,67,71]. Moreover, CD81 downregulation using siRNA in hepatoma cells abolishes HCV infection [15,64,72,73]. Although CD81 is normally expressed at the surface of primary hepatocytes and most hepatoma cell lines, it has been observed that HepG2, HH29 cells and also some sub-clones of Huh-7 cells do not express this tetraspanin. Interestingly, ectopic expression of CD81 in these non-permissive cell lines confers susceptibility to HCVpp and HCVcc infection [28,30,59,60,63,64,72,73,74,75], providing additional evidence for the importance of CD81 in HCV entry. Other studies have also shown that susceptibility of cells to HCV infection is closely related to CD81 expression levels [72,73,75] and that the ratio between cell surface levels of CD81 and SR-BI, another essential entry factor, also modulates HCV entry [61]. In addition, a study based on a mathematical model of HCV viral kinetics *in vitro* evaluated that between one and thirteen CD81/E2 complexes are necessary for HCV entry into hepatoma-derived cells [76].

### 3.2. Determinants in CD81/E2 Interaction

Blocking of HCV entry requires the disruption of E2-CD81 interaction, hence key domains in CD81 and E2 have been extensively studied. 

#### 3.2.1. Determinants in CD81

It was demonstrated early on that the E2 binding domain on CD81 is located in the large extracellular loop (CD81-LEL). Indeed, the use of recombinant soluble CD81-LEL to prevent sE2 binding to cell surface and to neutralize HCV infection has implicated this domain in E2 binding [16,77]. In addition, ectopic expression of CD81/CD9 chimeras (CD9 is a closely-related tetraspanin molecule) in HepG2 cells, which naturally do not express CD81, has confirmed the critical role of CD81-LEL in HCV entry [64]. Numerous studies have also shown that antibodies targeting CD81-LEL were able to neutralize infectivity ([28,42,46,65,71,78] and many other references). It has to be noted that, although they are not involved in a direct interaction with E2, other domains of CD81, namely SEL, TM3 and TM4 contribute to the functionality of CD81 in HCV entry [79,80].

The LEL epitope essential for CD81-E2 interaction has been identified. CD81 from African Green Monkey (AGM), which differs from the human CD81 by only four amino acids (T163A, F186L, E188K and D196E) is unable to interact with sE2. Interestingly, the expression of CD81 single mutants in KM3 cells showed that the F186L mutation prevented attachment of sE2 to the cell surface whereas the T163A mutation increased this binding, indicating that these residues might contribute to the CD81 ligand-binding ability and the tertiary structure of CD81 [81]. 

The crystal structure of CD81 LEL revealed that this domain displays a mushroom-like structure with two subdomains [48]. The first subdomain is composed of two antiparallel helices (A and E), that form the stalk of the mushroom as well as a third helix (B), which is connected to helix A by a short loop. The second subdomain is composed of two shorter helices (C and D) and is located at the top of the first subdomain. The two disulfide bonds stabilize this structure. It has been shown that this stabilization of CD81-LEL conformation is essential for the interaction with E2 [82] and that the E2 binding domain is likely in the variable C-D-double-helix subdomain. Indeed, Kitadokoro *et al.* have suggested that the highly conserved residues Ile^181^, Ile^182^ and Leu^185^ in the D-helix could be part of the E2 binding domain [48]. Subsequently, Ile^182^, Phe^186^, Asn^184^, and Leu^162^ were implicated using random mutagenesis studies. These mutations abolished CD81-LEL dimerization, suggesting that CD81 dimerization might play a role in CD81-E2 binding [83]. Very recently, these results have been confirmed and showed that, in addition to D-helix, the C-helix is likely also involved in E2 binding [84]. A nuclear magnetic resonance (NMR) spectroscopy study has pinpointed an extended 4‑residue turn involving a dynamic SNLFK motif that links helices C and E [85]. The authors suggested that the initial hydrophobic interaction between the dynamic SNLFK motif and the E2 protein could serve as a basis for a more substantial contact through the formation of a helical structure in the D-helix region. This conformational flexibility within the LEL domain might be required for CD81 to prime E2 for HCV entry. Conversely, the helical propensity of the residues comprising the SNLFK motif suggests the possibility of an induced helical structure upon E2 binding [85]. 

In the context of the full-length CD81 protein, some of the previously described mutations did not affect E2 binding [86]. In addition, the use of CD81 variants mutated for one of the amino acids that differ between hCD81 and _AGM_CD81 revealed that despite the absence of interaction with sE2, these variants support entry of HCVpp bearing envelope proteins from different genotypes [64,67,87]. Moreover, although the murine CD81 fails to interact with HCV glycoproteins or to inhibit HCV infection [16,87], it supports HCVpp and HCVcc infection [75,87,88]. Therefore, the link between CD81-E2 binding and CD81 ability to support HCV infection is still unclear. Moreover, cellular CD81 must be considered to better understand its interaction with the viral envelope proteins. Indeed, the use of an anti-CLDN1 antibody has suggested that the interaction between CD81 and CLDN1, another entry factor, might be important for binding to HCV E2 glycoprotein [89]. Searching for how to explain the capacity of some patients to clear HCV infection compared to other ones, CD81 polymorphism has been studied [90,91]. However, these studies failed to get a better understanding of residues essential for CD81-E2 interaction *in vivo*.

#### 3.2.2. Determinants in E2

The E2 glycoprotein plays a major role in the interaction between the virus and its major cellular entry factors. The CD81 binding region of E2 requires correctly folded E2 [35] and is comprised of discontinuous sequences that form the binding surface. Indeed, numerous studies based on the characterization of neutralizing antibodies, directed mutagenesis, analysis of cell culture adaptive mutations and modeling led to the identification of regions and residues of E2 that are potentially involved in CD81 interaction (Table 1). 

Neutralizing antibodies directed against E2 were studied to determine the ones that were able to prevent CD81/E2 interaction. Some of these antibodies target highly conserved conformational or linear epitopes in certain genotypes (reviewed in [92]). Thus, the study of cross reactivity of these antibodies on different genotypes and their ability to bind CD81/E2 complexes helped to determine crucial domains for CD81 binding (Table 1). The mapping of neutralizing epitopes revealed three domains in E2 proteins with distinct functions [93,94]. Neutralizing antibodies whose epitopes are in two of these domains can prevent CD81 binding to E1E2 complexes [94,95,96,97,98]. Other studies revealed that a region downstream of the hypervariable region 1 (HVR1) could be implied in CD81/E2 binding [41,77]. The AP33 antibody, which inhibits the interaction between CD81 and E2, raised particular attention due to its binding to a highly conserved E2 epitope comprising residues 412 to 423, with an exception for HCV genotype 5 [41,99]. A random peptide phage display approach identified residues Leu^413^, Asn^415^, Gly^418^ and Try^420^ to be highly conserved for AP33 recognition and thus, be part of the CD81 binding domain [100]. The mapping of other epitopes and their effects on CD81/E2 interaction led to define residues 480 to 493 (6/41a antibody epitope) and residues 544 to 551 (6/53 antibody epitope) as two potential discontinuous sequences for CD81 binding domain [77]. Escape mutants to neutralizing CBH-2 and HC-11 antibodies have highlighted the importance of two discontinuous regions, residues 425–443 and 529–535, in the structure required for virus binding to CD81 [97]. Additional epitope mapping studies have established the structure of parts of these domains. For example, co-crystallization studies of epitope peptides and Fab fragments of neutralizing antibodies helped defining the structure of the fragment from aa 412 to 423 recognized by AP33 and HCV1 antibodies [101,102,103] and the fragment from aa 434 to 446 recognized by HC84-1 and -27 antibodies [104]. However, these structures are still not sufficient to understand the complete organization of E2 and its interface with CD81.

**Table 1:** HCV E2 amino acids potentially involved in CD81 interaction.

E2 residues/regions ^a^	Tools ^b^	Assay	Authors
480–493/544–551	E2661	Blocking antibodies ^c^	[77]
517–535	E2715	Blocking antibodies	[105]
474–494/522–551	E2683	Modeling	[70]
407–524	E2660	Interstrain chimeras	[106]
412–423	E2660	Blocking antibodies	[41]
396–407/412–423	E1E2	Blocking antibodies	[41]
/432–447/528–535	VLPs ^d^		
HVRs/613–618	E2661	Deletions/Mutagenesis	[68]
HVR1	HCVpp	Deletion	[107]
G^436^WLAGLFY^443^	HCVpp	Mutagenesis	[108]
420, 527, 529, 530 and 535	HCVpp	Blocking antibodies/Mutagenesis	[99,109]
527, 529, 612–619	HCVpp	Mutagenesis	[110]
G451	HCVcc	Adaptive mutation	[111]
N415, 412–423	HCVcc	Blocking antibodies/Mutagenesis	[112]
V388, M405	HCVcc	Adaptive mutations to mCD81	[88]
DI and DIII domains	E2715	Modeling	[113]
529–535	HCVcc	Adaptive mutations to neutralizing antibodies	[97]
N415, HVR2 ^f^, IgVR ^g^	HCVcc	Deletion/adaptive mutation	[114]
H421	HCVpp	Mutagenesis	[115]
427, 428, 444 (Front layer)	E2 412–645	Crystallography Electronic microscopy	[84]
525, 527, 529, 530, 535 (CD81 binding loop)	E2 384–717	Mutagenesis	

^a^ Positions of amino acids in the polyprotein of reference strain H (GenBank accession no. AF009606). ^b^ E2661, E2715, E2683, E2660 are for sE2 ending at indicated positions. ^c^ Indicated E2 regions/amino acids correspond to the epitopes of blocking antibodies. ^d^ Virus-like particles produced in insect cells. ^e^ HVR1 includes residues 384 to 411. ^f^ HVR2 includes residues 460 to 485. ^g^ IgVR includes residues 570 to 580.

More precise E2 residues interacting with CD81 have been identified, as detailed in Table 1. The involvement of some has been confirmed with HCVpp and HCVcc derived E1E2 complexes. The different E2 hypervariable regions: HVR1, HVR2 and IgVR (for Intergenotypic variable region, which is conserved within a genotype) have been shown to modulate CD81/E2 binding, even if their role in this interaction is likely indirect [68,98,107,114]. Indeed, deletion of HVR1 increases binding to CD81, probably by exposing a CD81 binding domain [68,98]. However, a single adaptive mutation in E2 of a ΔHVR1 mutant virus, N415D, is able to fully restore HCV entry, probably by increasing E2 affinity for CD81, suggesting that HVR1 does not have a crucial role in CD81 binding [114], as initially suggested by Forns and collegues [105]. Interestingly, in another study using long-term passaging, the same adaptive mutation was observed and its selection led to an increased infectivity of HCVcc. Interestingly, this mutant virus was more sensitive to neutralization with CD81-LEL [112]. In addition, deletion of HVR2 or IgVR leads to a 50% decrease of CD81 binding and inhibits HCV entry, suggesting that these deletions impair E1E2 complex organization [114]. Another cell culture adapted JFH-1 mutant, G451R, which has a reduced dependency on SR-BI, showed an increased sensitivity to neutralization by soluble CD81 and enhanced binding of recombinant E2 to cell surface-expressed CD81 and CD81-LEL [111]. In another study, adaptation of HCVcc to mouse CD81 identified three mutations in envelope glycoproteins, one in E1 (L216F) and two in HVR1 of E2 (V388G and M405T), which were able to increase the interaction with both murine and human CD81 [88]. The authors have suggested that these mutations probably lead to an opening of the glycoprotein complex and that this “unlocked” structure increases exposure of the CD81 binding site. In turn, such conformational changes might permit the virus to utilize the weak E2 binder, mouse CD81 (mCD81), for its entry process. Additional residues have been suggested to be involved in CD81/E2 interaction, such as His^421^ for which the mutation abolishes CD81 interaction with HCVpp [115], as previously described [99]. Taken together, at least three E2 regions may directly interact with CD81; however, it remains to be defined if additional regions modulate CD81/E2 interactions.

A first modeling study, based on secondary structure prediction and fold recognition methods, proposed that three E2 segments bind CD81. These included a sequence from aa 474 to 494, another from aa 522 to 551 and a last one from residue 612 to 620 [70], which partially overlap sequences determined through the epitope mapping approach. A more recent model of organization of the tertiary structure of E2 has been proposed based on analogy with class II fusion proteins [113]. In this model, the E2 protein is organized in three domains: DI, DII and DIII. Most of the interaction determinants are predicted to be on DI surface, a domain organized in two beta-sheets containing each eight beta‑strands. The top sheet contains most of CD81 determinants while the bottom one only contains the Asn^556^ residue. In addition, CD81 is also predicted to bridge the surface of DI and DIII. DIII involved in CD81 interaction spans residues 613–618 [68,110]. It is of note that many E2 glycolysation sites are predicted to be located in DI, which would partially prevent recognition by neutralizing antibodies. These glycans might also have a role in modulating E2 binding to CD81 [116,117,118,119]. Very recently, the crystal structure of E2 ectodomain has been determined [84], it suggests that E2 might not be organized in three domains but has a globular structure containing many regions with no regular secondary structures. Indeed, the co-crystallization of E2 core bound to neutralizing antibody AR3C has revealed that E2 is composed of a central β sandwich flanked by front and back layers consisting of loops, short helices, and β sheets. Using site-directed mutagenesis and negative-stain electronic microscopy, Kong *et al.* demonstrated that CD81 likely interacts with several amino acids in the front layer of E2 (notably aa 427–430 and 442–444), and amino acids in the CD81 receptor binding loop (including aa 525 and likely the previously described aa 527, 529, 530 and 535 [99]) [84]. 

On the whole, it is still unclear which residues or sequences are involved in CD81/E2 interaction. Especially because it is difficult to discriminate between direct and indirect interacting residues, the latter enable binding to CD81 by an effect on E2 conformation. Moreover, most of the studies used sE2 or HCVpp in which the conformation of E2 is slightly different from the envelope glycoproteins in native HCV particles. E2 has been widely described as the major glycoprotein involved in CD81 binding; however E1E2 complexes play an important role in this interaction. Indeed, E2 conformation is influenced by its heterodimerization with E1 (reviewed in [120]). Importantly, co-expression of both E1 and E2 is essential for the production of infectious particles [28,42,46]. In addition, CD81 was shown to co-immunoprecipitate with E1E2 complexes rather than with E2 alone [40]. Interestingly, E1E2 complexes whose E1 cysteines have been mutated to disrupt disulfide bonds, showed an increased binding to CD81, demonstrating that E1 can modulate E2 binding ability to CD81 in the context of the HCVcc system [121]. Finally, it has been shown that more free thiol groups are present in HCVpp, as compared to HCVcc, indicating that arrangement of E1E2 complexes on HCVpp is probably different in HCVpp and HCVcc systems [122]. 

### 3.3. CD81 in HCV Species Tropism

HCV species tropism is quiet narrow since this virus naturally infects only humans and chimpanzees. It has been shown that sE2 binds to human but not to mouse, rat, or African green monkey CD81 proteins, suggesting that CD81 may be a factor in the species specificity of HCV infection [77,81]. However, CD81 cannot be the sole determinant, as transgenic mice expressing human CD81 (hCD81) fail to support HCV infection [123] and CD81 from tamarin, which is not susceptible to HCV infection, has been reported to bind sE2 [93,124]. In addition, ectopic expression of murine CD81 (mCD81) in CD81-deficient hepatoma cells restored to some extent permissivity to HCVpp and HCVcc [75,87]. Another study suggested that mutations in E1/E2 sequence enable the virus to adapt to mCD81, these mutations also enable the virus to use hCD81 even more efficiently [88]. However, the Huh-7w7 cell line, which does not express hCD81, can be infected by HCV when stably expressing mCD81 without any adaptation [75]. It has to be noted that tupaïa CD81 has been shown to be functional in HCV entry and tupaïa primary hepatocytes support full HCV infection [125,126,127,128,129], making of tupaia a new hope to develop a simpler animal model. 

Thus, CD81 contributes to, but does not alone define the species restriction; additional cellular factors are likely involved in restriction of HCV entry. Indeed, it has been reported that both hCD81 and hOccludin, another HCV entry factor, are partly responsible for species specificity [20] explaining the difficulty to develop animal models. Strikingly, combined expression of hCD81 and hOccludin in immunocompetent mice or humanized mice is sufficient to render them permissive to HCV infection [130,131].

### 3.4. Interplay with Other Entry Factors

CD81 was the first entry factor identified for HCV [16]. A subsequent study reported that SR-BI also bound to HCV E2 glycoprotein [17]. But very soon it became clear that although both CD81 and SR-BI are essential molecules required for HCV entry, expression of both proteins was not sufficient to confer HCV entry [28,59]. Indeed, the tight junction proteins CLDN1 and OCLN are also required alongside of CD81 and SR-BI to initiate virus infection [18,20], indicating that HCV entry is a multistep process. But the complexity of the mechanism of HCV entry does not stop there as it was shown that additional factors such as cellular kinases are also involved in viral entry [21,132] (Figure 1).

SR-BI was first identified as a potential HCV receptor because it binds sE2 through interactions with E2 HVR1 [17]. Later on, it has been demonstrated that HCV entry is strongly reduced in SR-BI knock-down hepatoma cells or by antibodies directed against SR-BI [59,61,133,134,135,136,137]. Moreover, SR‑BI overexpression enhances HCV internalization [136,138]. The kinetics of inhibition of HCV infection by anti-SR-BI antibodies were almost identical to those observed for anti-CD81 antibodies [137,139], indicating that SR-BI might act concomitantly with CD81. However, it is likely that HCV particles encounter SR-BI before CD81. Indeed, HCVcc can bind to CHO cells expressing SR-BI but not to CHO cells expressing CD81, suggesting that a first contact with SR-BI might be necessary for the particle to interact with CD81 [18]. Based on sequence differences between human and mouse SR‑BI, which does not bind E2, specific SR-BI protein residues required for sE2 binding have been identified [134]. Interestingly, SR-BI mutants with reduced binding to sE2 were also impaired in their ability to restore infectivity of a Huh-7.5 cell line in which SR-BI was knocked‑down. In addition, these SR-BI mutants were still able to form oligomeric structures and bind high-density lipoproteins (HDL) and mediate cholesterol efflux, suggesting that distinct protein determinants are responsible for the interaction with HDL and the HCV particle [134]. Indeed, SR-BI is a major receptor for HDL but also for native and modified lipoproteins and modified serum proteins. This receptor plays a crucial role in selective lipid uptake and bidirectional transfer of free cholesterol. Very recently, a structural model of SR-BI has been reported in which the exofacial domain of SR-BI shows a helical bundle where ligands bind, and a channel through which lipids can be translocated to the membrane bilayer [140]. Regarding the function of SR-BI in HCV lifecycle, many studies have shown that SR-BI likely plays a role at several steps of the HCV entry process: (i) during viral attachment through an interaction between SR-BI and the lipoprotein moiety of the particle; (ii) during binding through an interaction between SR-BI and E2; (iii) during internalization through its lipid transfer function [139,141]. It is believed that the interaction between HCV particles and SR-BI might lead to conformation changes in the E1E2 complex facilitating/priming its association with CD81.

The tight junction proteins CLDN1 (also Claudin-6 and -9) and OCLN have been identified as additional HCV entry factors [18,20,142,143]. Although for many years no interaction between E2 and CLDN1 could be shown [18,89,143], it has recently been proposed that E1E2 complexes can interact with the first extracellular loop (EC1) of CLDN1 whereas soluble E2 does not, indicating a critical role of E1 in the modulation of HCV binding to receptors [144]. Infection of human cell lines and primary hepatocytes can be blocked by specific antibodies directed against CLDN1 [89,145] and kinetics of inhibition with such antibodies showed that CLDN1 acts cooperatively with CD81 and SR-BI in HCV entry [89]. Similar to CLDN1, the initial binding steps of HCV to the cell membrane are not affected by OCLN gene silencing, suggesting that OCLN plays a role in a late entry stage [146]. In addition, OCLN depletion that impairs HCV entry does not perturb CLDN1 expression or localization, suggesting that both entry factors function separately during HCV infection [19,146]. Thus, every cell expressing human CD81, SR-BI, CLDN1 and OCLN is susceptible to HCV infection [20]. Several studies have highlighted a critical role for the CLDN1-EC1 in HCV entry [18,142,143,144,147] and additional Förster resonance energy transfer (FRET) and biochemical studies have demonstrated that CLDN1 associates with CD81 [148,149,150,151]. The underlying mechanism is still poorly described, although it has been suggested that the CD81/CLDN1 association is promoted by the recently discovered HCV entry factors EGFR and Ephrin A2 [21]. Mutation of residues 32 and 48 in CLDN1‑EC1 ablates its association with both CD81 and occludin as well as viral receptor activity, demonstrating an essential role for CLDN1/CD81 complexes in HCV infection [149]. Moreover, neutralizing anti-CLDN1 antibodies specifically disrupt CD81/CLDN1 interaction, suggesting that CD81-CLDN1 coreceptor complexes are critical for HCV entry. Thus, CLDN1 may potentiate CD81 association with HCV particles by way of E2 interactions. Indeed, anti-CLDN1 antibodies inhibit envelope glycoprotein E2 and virion binding to permissive cells. This suggests that the association of CLDN1 with CD81 enhances the binding of viral glycoprotein to the cellular HCV coreceptor complex which is required for viral internalization [89]. Determinants in CD81 necessary for the interaction with CLDN1, identified by mutations T149, E152 and T153 in CD81-LEL, abolish CLDN1/CD81 interaction without affecting E2 binding to CD81. This demonstrates that CD81 association to E2 or CLDN1 are two distinct functions and highlights a new functional domain of CD81 that is essential for virus entry [151]. 

The association of CD81 with CLDN1 might facilitate their coendocytosis. Unlike the tetraspanins CD82, CD63 or CD151, CD81 does not have the endocytic YXXØ motif in its C-terminal domain [152,153]. On the other hand, CLDN1 is known to be easily internalized by endocytosis upon different stimuli (reviewed in [154,155]). Interestingly, real-time imaging studies demonstrated that CD81 and CLDN1 are co-internalized in early endosomes. In addition, HCV infection or binding of both receptors by their antibodies increased CD81 and CLDN1 endocytosis, indicating that HCV might stimulate receptor trafficking to promote its internalization [156]. In addition, binding of HCVcc particles to hepatocytes or antibody mediated cross-linking of CD81 induced EGFR activation, which is required for HCV endocytosis. Indeed, EGFR ligands enhanced the kinetics of HCV entry by inducing the endocytosis of EGFR-CD81 complexes. Interestingly, anti-EGFR antibodies induced its internalization in the presence of EGFR kinase activation inhibitors, enabling EGFR to promote CD81 endocytosis, which is critical for HCV entry. These results indicate that EGFR-CD81 interplay and EGFR internalization are critical for HCV entry [157]. However, these results are in contrast with a study showing that stimulation with EGF has no effect on CD81 endocytosis [156]. Another study showed that some effectors of the MAPK pathway induced by EGFR activation are important for HCV entry [132]. In particular, the GTPase HRas seems to play an important role in HCV entry. Indeed, activation of EGFR activates HRas which then associates with CD81. This association is required for CD81 lateral diffusion allowing CD81/CLDN1 association. Thus, it is believed that EGFR promotes CD81/CLDN1 complex formation by inducing CD81 diffusion through HRas activation and facilitates their co-internalization with the HCV particle [132].

Since CLDN1 and OCLN are tight junction proteins, it has been first suggested that, after an initial interaction with SR-BI and CD81, HCV particles migrate from basolateral membrane to tight junctions (TJs) to meet CLDN1 and OCLN. This process has been described for the entry of Coxsackievirus B that requires initial binding to the decay-accelerating factor on the luminal cell surface, followed by lateral migration of the virus–receptor complex to TJ, where interaction with the Coxsackie Adenovirus Receptor occurs [158]. Since E2 binding to CD81 induces Rho GTPases signaling which in turn leads to a rearrangement of actin cytoskeleton [159], it was believed that this remodeling might allow the viral particle to migrate from the basolateral membrane to the TJ. In addition, CLDN1 localization in TJs was shown to correlate with cell permissivity to HCV infection [19,147]. However, increasing evidences suggest that HCV uses CLDN1 that is not located in TJs and that polarization limits HCV infection. Indeed, CD81, CLDN1 and SR-BI expression in normal and HCV-infected human livers is mainly limited to the basolateral–sinusoidal hepatocellular surface [160]. Moreover, deletion of CLDN1 C-terminal part involved in TJs association [161] does not inhibit HCV entry [18]. CLDN1 associates with CD81 at the basolateral membrane of polarized HepG2 cells, whereas pools of CLDN1 in TJ show a minimal association with CD81 [148,149]. In addition, disruption of TJs by calcium depletion of polarized HepG2 cells, as well as the depolarization of HepG2 cells induced by HCV replication, even increase HCV entry and cell-to-cell transmission [150,162]. It is worth noting that while some studies showed that EGFR activation upon HCV infection leads to an increase CD81/CLDN1 association, they were in contrast with FRET analysis studies showing that HCVcc infection has no effect on these complexes [148]. It has been suggested that CD81/CLDN1 complexes form naturally outside of TJs and are not influenced by HCV infection. This was also supported by the comparison of CD81/CLDN1 association in hepatocytes from healthy or infected donors that showed no difference [148]. Finally, a study observed neither rearrangement of actin cytoskeleton upon HCV entry nor migration of HCV particles to cell-cell contact areas after binding by live cell imaging, supporting the idea that TJs are not required for HCV entry [163].

The TJs protein OCLN was also described as an essential HCV entry factor [19,20], but its exact role is poorly described, essentially due to the lack of efficient inhibitors or antibodies targeting its extracellular domains. A most recent kinetic analysis used OCLN in which FLAG sequences were inserted. This study established that OCLN is involved in late entry events, after the involvement of both CD81 and CLDN1 [164], as previously suggested [146,165]. 

### 3.5. Priming Role of CD81

As described previously, CD81 is likely involved in both early and late steps of HCV entry. Whereas the role of CD81 in early entry steps as a binding factor and its interplay with CLDN1 have been extensively studied, little is known on its role at later entry steps. However, it has been shown that CD81 may induce conformational changes in E1E2 heterodimers to promote low pH-dependent fusion and endocytosis. Indeed, pretreatment of viral particles with CD81-LEL has shown to induce: (i) enhanced infectivity; (ii) altered recognition by conformation-specific antibodies to E1 and E2; (iii) E2 binding to liposomes; (iv) irreversible refolding/inactivation at low pH in the absence of a target membrane, and (v) fusion with the plasma membrane of permissive cells at acidic pH [166]. This demonstrates that CD81 likely primes envelope glycoproteins in late steps of HCV entry.

## 4. CD81 and Cell-to-Cell Transmission

Beside transmission by circulating particles referred to as cell-free infection, HCV particles can be transmitted directly into neighboring cells, so called cell-to-cell transmission (Figure 1). HCV cell-to-cell transmission was first suggested when infected cells foci were seen in infected human livers by RNA imaging analysis [167] and recently confirmed using the same approach [168]. Subsequent experiments of co-cultivation showed evidences of *in vitro* cell-to-cell transmission. Several studies demonstrated that CLDN1, OCLN and mainly SR-BI are required for cell-to-cell dissemination [169,170,171,172], while the involvement of CD81 is still controversial. Cell-to-cell transmission has first been described as a CD81-independent transmission route because HepG2 cells, which lack CD81 and are therefore not susceptible to cell-free HCV transmission, were infected when co-cultured with persistently infected human bone-marrow-derived B-lymphoblastoid cells [173]. Later on, naïve HepG2 cells were infected when co-cultivated with infected Huh-7.5 cells in presence of HCV neutralizing antibodies, which abrogated cell-free transmission, suggesting that CD81-independent routes exist in cell-to-cell transmission [174]. Additional studies came to the same conclusion [174,175,176]. In addition, *in vivo* studies showed that treatment with anti-CD81 antibodies prevented HCV infection when administrated before infection, but was unable to block HCV spread therapeutically soon after infection [65]. However, other studies demonstrated that cell-to-cell transmission is a CD81‑dependent process because CD81-deficient cells were refractory, whereas ectopic expression of CD81 renders them susceptible to HCV cell-to-cell transmission [74,169,177]. Interestingly, using simultaneously two neutralizing antibodies targeting both CD81 and HCV envelope proteins, a recent study highlighted the coexistence of CD81-dependent and CD81-independent cell-to-cell transmission [172], as suggested by Brimacombe and colleagues [169]. This treatment reduced cell-to-cell transmission only by 60%, raising the possibility that a CD81-independent cell-to-cell transmission route accounts for the residual 40% [172]. It has to be noted that in this study, Catanese *et al.* also demonstrated that 48h of co-culturing of CD81^+^ cells with target cells lacking CD81, promotes transfer of CD81 to target cells. It is therefore possible that controversial results on CD81-involvement in cell‑to-cell transmission are due to some transfer events of CD81 from donor cells to target cells in the different experimental conditions. In addition, differences in cell lines and/or viral strains cannot also be excluded, as described for HIV [178]. 

On the whole, mechanisms of HCV cell-to-cell transmission are still unclear. The identification of the precise mechanisms should also define the contribution of CD81 in this process. Interestingly, it has been very recently demonstrated that exosomes (which are enriched in tetraspanins such as CD81 [179]) from HCV-infected cells were capable of transmitting infection to naïve human hepatoma cells, in the presence of neutralizing antibodies [180].

## 5. Association of CD81 with EWI Proteins

As described previously, CD81 belongs to the tetraspanin family, which are proteins that interact with each other and with other transmembrane proteins, forming extended cholesterol-rich complexes on the cell surface, called tetraspanin-enriched microdomains (TEMs) or tetraspanin webs (reviewed in [181]). In these domains, tetraspanins form primary complexes with a limited number of proteins called tetraspanin partners. These primary interactions are direct, highly specific and occur at high stoichiometry. CD81 was shown to interact with numerous partners such as CD19 [182], CD4 [183], CD8 [183], integrin α4β1 [184] and MHC-II [185]. In most cell lines, CD81 is also associated with a high stoichiometry with EWI-F (also called CD9P-1), EWI-2 and EWI-2wint (for EWI-2 without its N-terminus) [186,187,188,189,190,191], three members of the EWI family, a small Ig-domain family whose members have a single transmembrane domain and several extracellular Ig-domains with a conserved EWI (Glu‑Trp-Ile) motif, as well as a very short cytosolic tail [190]. Interestingly, it has been demonstrated that the direct interaction between tetraspanins and their partner proteins modulates their functions. Indeed, CD81 functions in cellular processes and infectious diseases were shown to be affected by association with EWI proteins. For instance, EWI-2 modulates CD81-dependent cell migration [192,193,194] and EWI-F/CD9P-1 acts as a negative regulator of *Plasmodium* infection by interacting with CD81, which is essential to *Plasmodium* liver infection [195]. One of the best examples is the modulation of CD81 functions by its associated partner EWI-2wint. EWI-2wint is a cleavage product of EWI-2 in which the first of the 4 extracellular Ig-domains is cleaved off, which was identified a few years ago [189]. This shorter protein forming heterodimers with EWI-2 [80] still interacts with CD81, and can be found in most cell lines expressing EWI-2 but not in hepatocytes. Importantly, in contrast to full-length EWI-2, EWI-2wint inhibits HCV infection by inhibiting viral entry [189]. Thus, in addition to the presence of specific entry factors in the hepatocytes, lack of this specific inhibitor may contribute to the hepatotropism of HCV. The determinants for the interaction of EWI-2/EWI-2wint and CD81 have been identified [80] and the mechanism by which EWI-2wint blocks HCV entry has been recently elucidated [177]. Indeed, EWI-2wint restricts HCV entry by blocking E2-CD81 interaction [189] and by promoting the formation of CD81 oligomers that diffuse more slowly in the plasma membrane [177]. These oligomers are recognized by a monoclonal antibody that poorly neutralizes HCV infection, indicating that CD81 oligomers are not used during HCV entry. Hence, by changing the membrane partitioning of CD81 molecules on the cell surface and thereby their membrane diffusion, EWI-2wint impedes the interaction between CD81 and E2 envelope glycoproteins and thus blocks HCV entry [177]. 

## 6. Significance of CD81 Membrane Diffusion in HCV Entry

During their entry and cell-to-cell transmission, many viruses take advantage of cellular mechanisms that normally drive the movements of proteins and lipids on the cell surface (reviewed in [196]. It is therefore essential to focus attention on the membrane dynamics of virus entry factors. The dynamics and partitioning of CD81 were probed using single-molecule tracking (SMT), a technique based on labeling of a low number of molecules allowing individual molecules to be optically isolated and their position accurately determined [177,197]. With this technique, the position of proteins can be determined frame by frame, their trajectories reconstructed and the apparent diffusion coefficient calculated using a linear fit to the mean squared displacement *versus* time plots. As described for CD9, another tetraspanin [198,199], CD81 molecules display three modes of membrane diffusion: (i) pure Brownian diffusion that corresponds to CD81 molecules with high diffusion coefficients; (ii) pure confined diffusion that corresponds to CD81 molecules with low diffusion coefficients; (iii) diffusion with different combinations of Brownian and confined modes referred to as “Mixed trajectories”. Simply, CD81 molecules, as well as other tetraspanins such as CD9, can be seen as highly dynamic proteins distributed along the whole cell surface but are enriched in areas of the membrane to form stable platforms in position and shape although in permanent exchange with the rest of the membrane [181,198]. Interestingly, two studies demonstrated that the respect and the balance of these dynamic exchanges in the cell membrane are essential to the process of HCV entry [177,197]. Indeed, the study on CD81 membrane behavior in cells expressing EWI-2wint, which inhibits HCV entry, demonstrated a striking effect on membrane dynamics of CD81 [177]. The diffusion of CD81 was largely reduced upon EWI-2wint expression due to two principal effects. First, the number of pure confinement largely increased, due to the trapping of CD81 molecules within membrane domains. Second, the diffusion rate of CD81 Brownian molecules significantly decreased because CD81 diffused in larger complexes, which were formed thanks to EWI-2wint interacting more strongly with CD81. In addition, EWI-2wint increased the colocalization of CD81 with CLDN1. Hence, this study gave new insights on the mechanism by which HCV enters into its target cells, namely by exploiting the dynamic properties of CD81. The significance of CD81 membrane diffusion in HCV entry was also demonstrated by Harris and colleagues [197]. They showed that the mobility of membrane CD81 is correlated with the level of HCV infection [197]. Using fluorescence recovery after photobleaching (FRAP) and SMT experiments, they reported that CD81 mutated in its C-terminal domain was less mobile than wild type CD81 expressed in non-polarized HepG2 cells. In parallel, results of infection experiments of these two cell lines showed that infection was inhibited in cells expressing mutated CD81. A second observation allowed to connect the dynamic properties of CD81 to its capacity to mediate the HCV entry. Indeed, a reduced diffusion of CD81 and an increased frequency of confinement were observed in HepG2/CD81 polarized cells [197], in which HCV entry is inhibited compared to that observed in non-polarized cells [150]. In contrast, activated macrophages promote infection of polarized cells through the production of TNFα that increases membrane diffusion of CD81 [200].

Taken together, these studies demonstrate that events limiting membrane diffusion of CD81 (*i.e.*, the presence of EWI- 2wint, mutation of the cytoplasmic domain of CD81 or cell polarization) interfere with the entry of HCV, suggesting that CD81 molecules that freely diffuse and are therefore not engaged in static microdomains are used by HCV during its entry step (Figure 1).

## 7. CD81 and HCV Replication

CD81 is a key player in the entry process of HCV lifecycle. However, some studies suggest a role for CD81 in late steps of HCV lifecycle. It was first suggested that CD81 might be involved in HCV secretion. A study showed that CD81 was necessary for E1E2 glycoproteins maturation and secretion into exosomes [201]. Because this study used CHO cells overexpressing E1E2 and/or hCD81, it is therefore possible that the cell line used as well as the absence of the whole HCV particle assembly process did not mimic what really occurs. Interestingly, these authors showed that HCV RNA is associated with exosomes in infected patients, which is in line with recent studies [180,202]. A later study used HCV subgenomic replicons and cell lines expressing different levels of CD81 and demonstrated that CD81 is not only required for entry, but also for HCV RNA replication. In this study, low CD81 expression level affected slightly HCV entry but had a high impact on RNA replication while it did not impair HCV protein synthesis [203]. The precise mechanism of the involvement of CD81 during replication needs to be elucidated further. Conversely, HCV RNA replication downregulates CD81 cell surface expression, reducing the total CD81 protein level and retaining it intracellularly, thereby interfering with HCV reinfection [204]. The authors used a modified JFH-1 strain containing a blasticydin-resistance cassette, allowing the generation of highly‑replicating HCV stable cell lines producing high amount of HCV RNA and proteins that became resistant to the re-infection by HCVcc or HCVpp, due to their lack of CD81 at the cell surface. Ke and Chen also proposed that in response to robust HCV RNA replication, cells activate pathways downregulating the expression and intracellular localization of CD81, leading to impaired ongoing virus transmission and persistent infection, thus controlling viral propagation [204]. Thus, HCV superinfection exclusion might not be related to the selection of a cell population expressing low levels of CD81, as previously suggested [205].

## 8. Role of CD81 in Immune Response to HCV Infection

**Figure 4:**
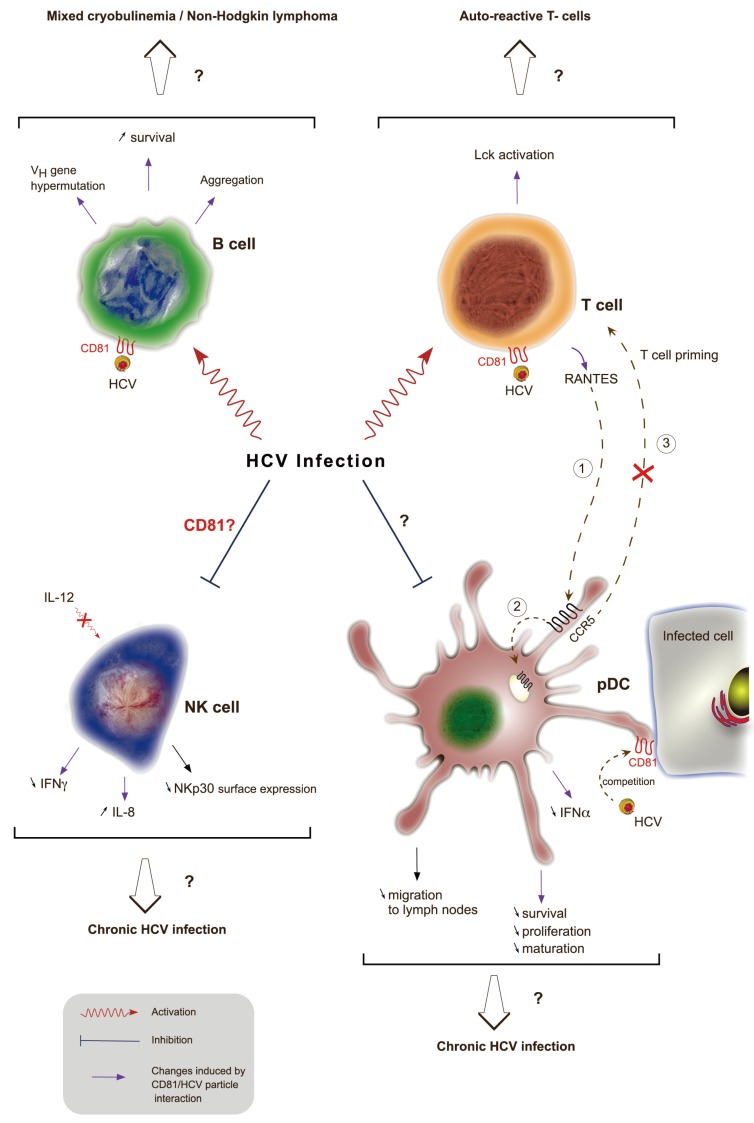
Role of CD81 in disrupting HCV-induced immune functions. During HCV infection, viral particles interact with CD81 at the cell surface of different immune cells through E2 envelope glycoprotein. This interaction leads to B cell activation, aggregation, increased survival and triggers V_H_ gene hypermutation. This might result in mixed cryobulinemia and non-Hogkin’s lymphoma. The interaction of E2 with CD81 on T cells also induces signaling as shown by activation of the protein tyrosine kinase Lck. This activation could be responsible for the presence of auto-reactive T cells in chronically infected HCV patients. This interaction leads to the production of RANTES, a ligand for the CCR5 receptor. RANTES ligation to CCR5 (1) at the surface of pDCs is responsible for its internalization (2) and thus prevents pDCs migration from infected tissues to lymph nodes and thereby priming of T cells (3). Direct interaction of E2 with CD81 at the surface of pDCs leads to a decreased secretion of IFN-α and impaired maturation, proliferation and survival of these cells. The interaction of free HCV particles competes with the recognition of infected cells through domains enriched in CD81. Finally, HCV infection causes an impairment of NK cell functions. The role of CD81 in NK cell-altered function is controversial and could lead to a decreased IL-12-dependent IFN-γ secretion and an increased secretion of IL-8. Independently of CD81, HCV infected cells interact with NK cells and decrease NKp30 cell surface expression in NK cells. On the whole, impairment of pDCs and NK cell functions might explain the establishment of chronic HCV infection.

HCV infection has been reported to be associated with immune system disorders (reviewed in [206], such as mixed cryoglobulinemia [207] or B cell non-Hodgkin’s lymphoma [208]. Cryoglobulins are immunoglobulins that become insoluble at a temperature below 37 °C. Mixed cryoglobulinemia correspond to high concentrations of cryoglobulins containing a mixture of monoclonal and polyclonal or a mixture of polyclonal immunoglobulins. Up to 90% of unexplained mixed cryobulinemia are associated with chronic HCV infection and up to 60% of chronically infected patients develop mixed cryobulinemia (reviewed in [209]. However, little is known about the mechanisms leading to these B cell lymphoproliferative diseases. Engagement of CD81 by HCV envelope proteins induces *in vitro* B cell aggregation and protein tyrosine phosphorylation [210], a hallmark of B cell activation (Figure 4). In addition, chronic HCV infection is associated with an increased activation of naïve and memory B cells [211]. E2-CD81 interaction on B cells is also associated with V_H_ gene hypermutation through the upregulation of cytidine deaminase and error prone DNA polymerase ζ [212]. B cell viability is also increased by CD81 engagement with E2 as this interaction up-regulates anti-apoptotic proteins from the Bcl-2 family [213]. Altogether, it is possible that these CD81-E2 interactions contribute to HCV-associated B cell diseases. However, another study showed that B cell expansion in the peripheral blood of HCV infected patients is not associated with antigen‑mediated activation and differentiation. Other mechanisms such as an altered homeostatic regulation of mature B cells or increased levels of circulating cytokines may promote increased numbers of circulating B cells [214].

On B cells CD81 is part of a complex with CD21, CD19, and IFITM1 (Leu13) [215]. This complex reduces the threshold for B cell activation via the B cell receptor by bridging antigen specific recognition and CD21-mediated complement recognition. If the CD81/E2 interaction seems to play a rather controversial role in disturbing immune cells functions, HCV is also able to disturb innate immune response in host cells. Indeed, the ISG (Interferon Stimulated Gene) from the IFITM family, IFITM1, expressed upon IFN-α induction, is downregulated by HCV. Although IFITM1 was shown to be able to decrease HCV replication by an unknown mechanism [216], IFITM1 appeared to be able to interact with CD81 and OCLN and thus disturb the HCV entry process [217]. 

B cell-associated disorders are not the only immune disorders related to HCV infection (Figure 4). Disturbance of Natural Killer (NK) cells have showed contradictory results. Early reports demonstrated that cross-linking of CD81 at the surface of NK cells by E2 altered their function. This included down-regulation of their activation through cytoskeleton rearrangement, by inhibiting CD16‑mediated tyrosine phosphorylation and by decreasing IFN-γ production [218,219,220]. These results support the observation that PBMC from healthy individuals exposed to HCV containing serum from infected patients lead to an inhibition of NK-cells functions [221]. This inhibition of NK cells function could partly explain the establishment of HCV chronic infection However, more recently, a report using intact HCV particles has shown that NK cell functions were not altered by E2-CD81 interaction [222]. These contradictory results are probably due to the differences in E2 conformation between sE2 and E2 on viral particles. The study of Yoon *et al.* is in agreement with an additional recent study, which showed that CD81/E2 interaction was not responsible for altered NK cells functions *ex vivo*. Indeed, the study demonstrated that infected Huh7.5 cells did not prevent NK cell function when blocked by either anti-CD81 antibodies or by E2. Specifically, they showed that a direct contact with infected cells is required leading to downregulation of NKp30 on the surface of NK cells, probably through the upregulation of a NKp30 ligand on the surface of HCV infected cells [223]. Moreover, NK cells just exposed to sera from infected patients or to HCVpp were activated [224]. HCVcc also modulated the pattern of cytokines produced by NK cells leading to reduced antiviral activity, they strongly reduced the IL-12-dependent IFN-γ production upon CD81 engagement while increasing IL-8 production, a cytokine which has no effect on HCV infectivity. This was supported by a study in which co-culture of NK cells, pDCs and infected Huh-7 cells enabled NK cells to produce IFN-γ through IL-12 stimulation, but only through pDCs-produced IFN-α [225,226].

While HCV-induced activation of NK cells results in reduced IL-12 production, co-engagement of CD81 with E2 and the T cell receptor (TCR) complex leads to the activation of TCR_γδ+_ T lymphocytes, which could result in autoimmune phenomena through activation and proliferation of auto-reactive T cells [227,228,229]. Interestingly, T cell co-stimulation by E2 interacting with CD81 was dependent on the activation of the protein tyrosine kinase Lck and downstream signalization through TCRζ [230].

Plasmocytoid Dendritic Cells (pDCs) functions were shown to either be altered [231,232,233,234,235] or unmodified [236,237,238] in HCV chronic infection. Core protein, NS3, NS4 and NS5 were first shown to impair pDCs functions, but more recently, the glycoprotein E2 was reported to be involved in altered pDC trafficking from the liver to lymph nodes. Indeed, cross-linking of CD81 with E2 on CD8^+^ T cells leads to release of chemokines such as RANTES whose binding to the CCR5 receptors on the surface of pDCs is responsible for its internalization. However, CCR5 is also needed for migration of pDCs to lymph nodes through the binding of its chemotractant ligand, CCL21. Thus, pDCs accumulate in the liver of chronically infected individuals and are unable to prime T cells [239]. In addition, pDCs are able to produce IFN-α [240] and IFN-γ [226] after cell-cell contact with HCV infected hepatoma cells but not in contact with free HCV particles, which inhibit IFN-α production by pDCs. It is likely that HCV RNA mediates pDCs signaling through TLR7 receptor [241]. This is in agreement with the fact that CD81 cross-linking with immobilized E2 inhibits IFN-α secretion by pDCs and impaired their maturation, proliferation and survival [242]. Finally, it has been shown that CD81- and CD9-enriched membrane microdomains are implied in pDCs interaction with infected hepatoma cells and are likely involved in endocytosis of HCV components such as viral RNA. Therefore, circulating infectious HCV particles in chronically infected patients could explain the decreased pDCs response because of the interaction between CD81 expressed on pDCs with E2 expressed on HCV particles [240].

## 9. Conclusions

In conclusion, the tetraspanin CD81 is a central regulator of HCV lifecycle. By an interplay with the other HCV entry factors, and likely because of its membrane diffusion properties, it acts as an organizer of the HCV entry factor complex thus controlling the initiation of infection. Although, precise mechanisms still need to be defined, CD81 might also play an important role in HCV RNA replication, dissemination and host response. This makes it a potential therapeutic target, as evidenced by the *in vivo* use of anti-CD81 antibodies [65]. However, CD81 is involved in a large number of cellular responses, thus the use of such a therapeutic strategy in humans could lead to side effects. Nevertheless, since CD81 associates with CLDN1 to form complexes essential to viral entry, a promising therapeutic strategy would be to develop tools that specifically target these complexes or by targeting the molecules involved in their regulation (as reviewed in Zona *et al.* [243]). Such a strategy would be particularly appropriate to avoid systematic reinfection in liver transplant patients.
